# Trigeminal Postherpetic Neuralgia: From Pathophysiology to Treatment

**DOI:** 10.1007/s11916-023-01209-z

**Published:** 2024-01-23

**Authors:** Christy S. Niemeyer, Michael Harlander-Locke, Andrew N. Bubak, Rachael Rzasa-Lynn, Marius Birlea

**Affiliations:** 1 Department of Neurology, University of Colorado Anschutz Medical Campus, Aurora, CO USA; 2 Department of Anesthesiology, University of Colorado Anschutz Medical Campus, Aurora, CO USA

**Keywords:** Trigeminal, Postherpetic, Neuralgia, Pain, Zoster, Virus

## Abstract

**Purpose of Review:**

Trigeminal postherpetic neuralgia (TG-PHN) is a neuropathic pain condition complicating herpes zoster (HZ) attributed to the trigeminal nerve. It poses significant challenges due to its persistent and debilitating nature. This review explores the clinical characteristics of TG-PHN, analyzes its pathophysiological underpinnings, and addresses existent and potential therapies.

**Recent Findings:**

TG-PHN is one of the most common and complex PHN locations. It has distinguishing clinical and pathophysiological characteristics, starting with viral triggered injuries to the trigeminal ganglion (TG) and peripheral tissue and involving the ascending and descending brain modulation pathways. Current therapies include vaccines, oral and topical medications, and interventional approaches, like nerve blocks and neurostimulation.

**Summary:**

This review covers TG-PHN’s clinical and physiological components, treatment options, and potential future targets for improved management. By exploring the complexities of this condition, we aim to contribute to developing more effective and targeted therapies for patients suffering from trigeminal PHN.

## Introduction

Varicella zoster virus (VZV) is an alphaherpesvirus that causes varicella upon primary infection, after which it becomes latent in neurons of trigeminal ganglia (TG) and dorsal root ganglia across the entire neuraxis. Over 90% of the world’s population has been infected with the virus, typically in childhood [1]. As VZV-specific immunity wanes, such as seen with normal aging or immunosuppression, VZV can reactivate to cause herpes zoster (HZ), a dermatomally distributed painful rash, with more than 1 million cases annually in the USA [2]. Most cephalic zoster (58%) affects the trigeminal nerve [3]; its first division, herpes zoster ophthalmicus (HZO), accounts for 2.5–20% of all HZ cases [4].

Zoster’s most common complication is postherpetic neuralgia (PHN), which is the persistent HZ-associated pain that can last for months to years after the rash has cleared, severely impacting the function and quality of life in affected individuals [4]. HZ occurring in the ophthalmic distribution (reactivation from TG) poses a higher risk of developing PHN (termed *trigeminal postherpetic neuralgia*; TG-PHN) compared to HZ in any other distribution (cervical, thoracic, lumbar, or sacral) [2, 5, 6]. TG-PHN is defined as a unilateral facial pain caused by HZ, persisting or recurring for at least 3 months after HZ, in the distribution of one or more branches of the trigeminal nerve; it may emerge in continuation of the acute zoster pain or develop after a painless interval [7–9]. The PHN resolves within 1 year in the majority of patients (up to 78%) but may persist for 2–10 years in 22–46% and indefinitely in others [10–12].

Here, we aim to present an inclusive overview of the current understanding of TG-PHN, focusing on elucidating potential cellular and physiological mechanisms underlying its development and persistence. Due to the challenges of TG-PHN’s clinical management, understanding its pathogenesis becomes imperative and may offer valuable insights into more targeted and effective treatment approaches.

## Risk Factors to Develop PHN After HZ

Many risk factors are associated with the development of TG-PHN following HZ, including demographics and zoster features.

### Demographics

The incidence of PHN increases with advancing age [5, 9] and ranges from 1.6 cases/100,000/year before 10 years of age to 228.5 cases/100,000/year in patients 71 years of age and older [2]. Moreover, PHN complicates over 40% of HZ cases in individuals over 60 years of age, and it is approximately five times more common in those aged 65 and above, compared to the rest of the population [5, 13]. While the average age in HZO was reported at 58 years old [14], patients who developed PHN after HZO were found to be older, 80 years of age being a noted cutoff [15]. HZ and PHN seem to display gender/sex and race/ethnicity differential frequencies, higher in women than in men, higher in whites than black, Asian, and Hispanic individuals [2].

### Location

**Fig. 1:**
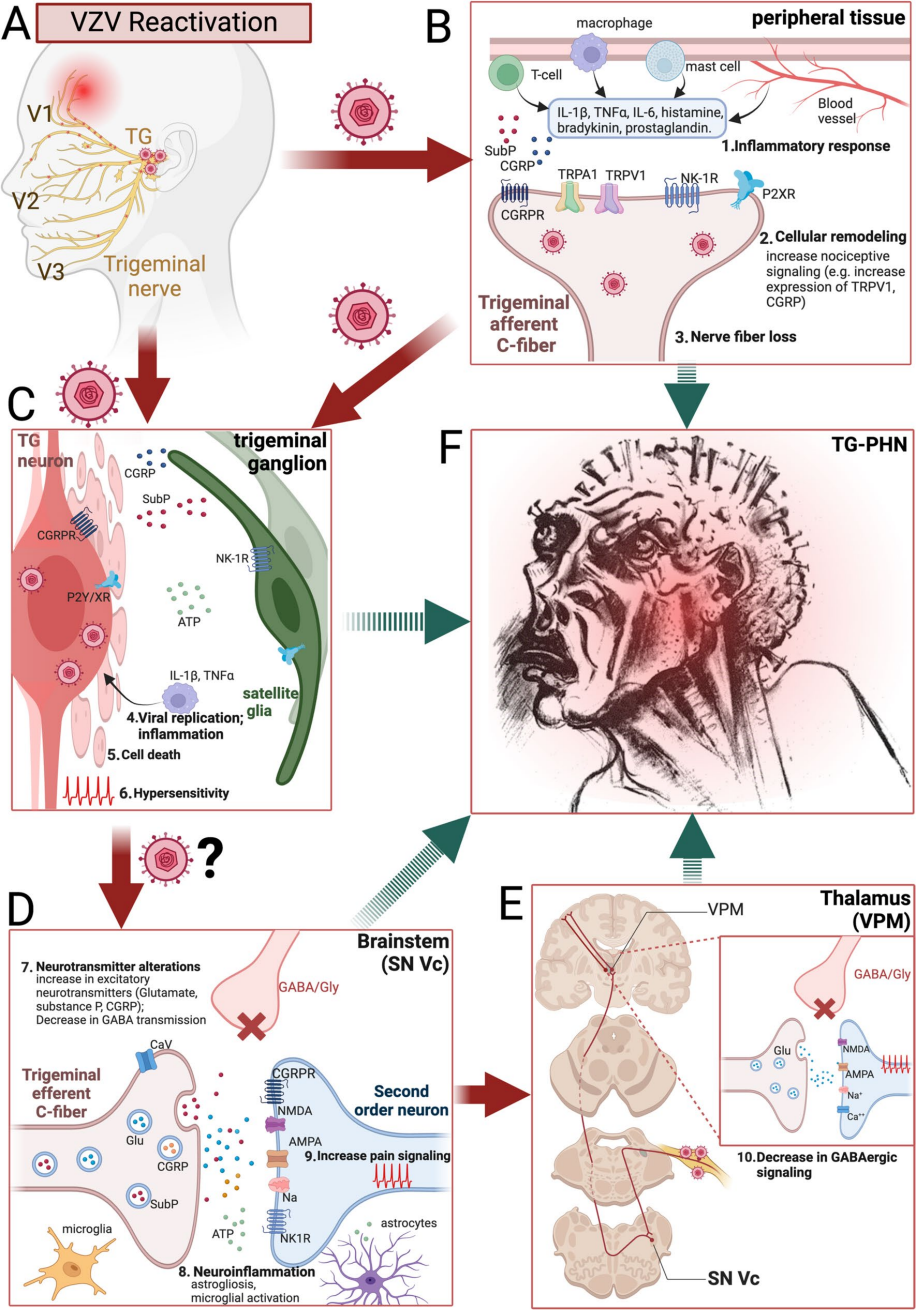
Comprehensive overview of pathophysiology of trigeminal postherpetic neuralgia (TG-PHN). **A** VZV reactivation predominately occurs from V1 distribution of the trigeminal ganglia. **B** Representation of cellular response at the site of reactivation highlighting the (1) inflammatory response involving macrophages, T cells, mast cells, and blood vessels; (2) changes in trigeminal afferents, particularly alterations in nociceptors influencing pain signaling such as TRVP1/TRPA1, CGRP, NK-1R, and purinergic receptors P2XR, along with associated ligands like substance P (SubP) and CGRP, ultimately resulting in (3) loss of nerve fibers and sensory innervation. **C** Representation of VZV reactivation and (4) viral replication of VZV and associated inflammation in the trigeminal ganglia. This leads to (5) cell death and can cause (6) hypersensitivity in the ganglionic neurons, increasing pain signals to the brainstem. Hypersensitivity can also be attributed to increases in neurotransmitters CGRP, SubP, and ATP. **D** Trigeminal ganglionic neurons transmit signals to the spinal trigeminal ganglia, specifically the caudal subunit (SNVc), which transmits nociception and thermal sensation. Neuroinflammation, including (8) astrogliosis and microglia activation, can cause disruption in the microenvironment within the brainstem. The increase of neurotransmitters, shown in C, is also released onto second-order neurons. Along with a decrease in inhibitory GABAergic signaling, together leads to (9) an increase in pain signaling. **E** Represents the pain signaling pathway from SNVc to the ventral posteromedial nucleus (VPM), where it has been shown to decrease GABAergic signaling in the thalamus, increasing pain signaling relayed to cortical structures. **F** These pathophysiological changes ultimately lead to the wide array of pain qualities associated with TG-PHN as represented in this artistic depiction reprinted with permission from Ref. [26]. Figure 1 was created with BioRender.com

PHN occurs more often after trigeminal zoster, prevailing HZ single-level location [16•], and HZO was associated with over twice the risk of PHN, compared with non-ophthalmic zoster [17]. The trigeminal division predominantly affected by PHN (over 75%) is the ophthalmic (V1), compared to the second or third divisions (V2, V3) [9, 18] (Fig. 1A). HZ involving nasociliary nerve (37.5% of HZO) was not associated with increased risk of PHN [15]*.*

### Rash Severity

In a cohort of HZO patients, 28% exhibited a severe skin eruption significantly associated with a higher occurrence of PHN [19]. Keratitis, conjunctivitis, and/or uveitis during HZO may enhance the risk for developing PHN, although not conclusively [5, 15]. A substantial VZV load in the nerve and ganglion of the most affected dermatome, notably ophthalmic, can lead to the heightened incidence of HZ and subsequent PHN in this area [18].

### Pain Severity

Severe HZ pain at presentation, including HZO, was independently associated with risk of PHN [15, 20]. However, when examining the emotional or cognitive response to pain (pain catastrophizing) in the acute phase of HZ, one report found that response during the initial presentation to be unrelated to the PHN development [21].

### Zoster Sine Herpete (ZSH)

Zoster without rash (sine herpete) can also occur. In ZSH, pain is more intense than canonical HZ with skin lesions, at presentation and long-term follow-up. Patients with post-ZSH pain followed up to 11 months had more prolonged and severe pain than those with PHN post-typical HZ and higher opioid use, which may also suggest greater pain burden in ZSH cases [22].

### Laboratory Findings and Comorbidities

VZV DNA was detected in the peripheral blood mononuclear cells months to years after the resolution of the zoster rash in patients with PHN, potentially reflecting a higher viral burden in the ganglia than during actual latency [23]. Furthermore, 3–12 months following an episode of HZ, especially HZO, individuals older than 50 are at higher risk for stroke than the general population [4, 24, 25]. Possible correlations with TG-PHN may be worth studying.

## Clinical Features of Trigeminal PHN

### Pain

TG-PHN patients experience a varied array of pain qualities, including deep, burning, continuous or paroxysmal, lancinating, or even diffuse piercing (artistic depiction of a patient’s own TG-PHN pain, Fig. 1F) [26]. Other sensory disturbances often linked with PHN are paresthesia, dysesthesia, hyperalgesia, and itching [18]. Chronic trigeminal postherpetic pain can exhibit different phenotypes, i.e., meeting the criteria for hemicrania continua [27] or SUNCT [28] but identifiably caused by VZV reactivation.

### Numbness

Numbness is a common symptom in TG-PHN. TG-PHN patients show sensory loss and allodynia, but not universally, others display thermal and/or pinprick hyperesthesia instead [9]. A comparative study revealed that numbness and paresthesias are significantly more prevalent in PHN affecting the face than in truncal PHN (where there is more prominent burning and allodynia), suggesting greater nerve degeneration in trigeminal cases [29••].

### Postherpetic Pruritus (PHP)

PHP is characterized by chronic itching and can be frequently experienced by PHN patients, especially in TG-PHN compared to other areas (43% vs. 25%) [16•]. In a notable case report, a 39-year-old woman experienced severe painless postherpetic itch after HZO, leading her to scratch through her skull into the brain within a year. Zoster recurred after 15 months in the same dermatome, causing lancinating pain and allodynia, diagnosed as TG-PHN. The itching and scratching continued, raising questions about losing peripheral sensory neurons and mechanisms underlying neuropathic itch [30]. It is unclear why higher rates of PHP are associated with the TG-PHN. However, several factors, including viral load and nerve damage, may play a role [16•].

### Trigeminal Trophic Syndrome

Trigeminally innervated tissue damage can occur months or years after zoster, e.g., ophthalmic [31] or mandibular distribution [32], and can even be a complication of interventional treatment for TG-PHN [33].

### Cerebral Symptoms

Patients with TG-PHN have a high prevalence (~ 40%) of insomnia, anxiety, depression, and cognitive difficulties. TG-PHN patients also report chronic fatigue, weight loss, and anorexia. Notably, patients with TG-PHN were more often severely depressed than those with thoracolumbar PHN [29••]. Allodynia in PHN, indicating central sensitization, is associated with pain intensity and was correlated with anxiety and depression [34]. All these complications significantly impair patients’ ability to function, highlighting the multifaceted challenges associated with TG-PHN [35]*.*

## Physical Exam and Testing

The physical examination and testing of TG-PHN are pivotal aspects of the diagnostic and management process, offering valuable insights into the nature and severity of the condition. In a study of 18 patients with TG-PHN pain, there were significant sensory changes to the ipsilateral side compared to the unaffected side, even in nearby trigeminal branches that did not experience pain [36]. Neurophysiological abnormalities, such as delayed blink reflex and reduced specific fiber amplitude, were observed on the side with pain compared to the unaffected side in PHN patients. Quality of pain, rather than allodynia, correlated with specific neurophysiological parameters [37].

**Fig. 2:**
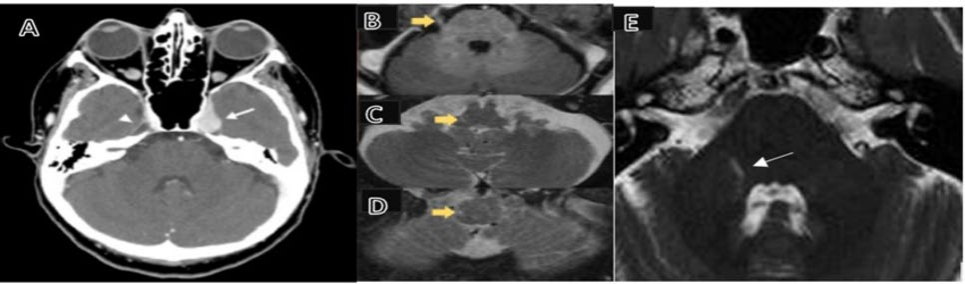
Imaging findings in trigeminal HZ and PHN. **A** Tumor-like enhancement (arrow) of the trigeminal ganglion, representing chronic VZV ganglionitis in a patient with ipsilateral PHN. **B**–**D** Axial T2 MRI hyperintensity of the right trigeminal nerve root, pons, and medulla in a patient with acute right V1 zoster (yellow arrows). **E** “Trigeminal pontine sign”—axial T2 MRI hyperintensity in a patient with right V1 trigeminal PHN (arrow). Reproduced from Refs. [38–40] (modified)

Head imaging can be revealing in certain cases, like a VZV trigeminal ganglionitis (enhancing TG mass on CT) in a patient with chronic ipsilateral trigeminal distribution pain without rash (Fig. 2A) [38]. A “trigeminal pontine sign” may suggest herpetic etiology for some cases of trigeminal neuralgia (TN) and a similar T2 hyperintensity can be seen in the area of spinal trigeminal nucleus in ipsilateral trigeminal zoster (Fig. 2B–E) [39, 40]. Additionally, MRI showed thickness alterations in brain areas related to sensory, motor, and cognitive functions in PHN patients, as well as abnormalities in the ascending and descending modulation pathways; those can include smaller thalamus and amygdala volumes and weaker functional connectivity between periaqueductal gray and anterior parts of pain matrix [41, 42•]. The above neuroanatomical alterations, among others such as white matter changes, may contribute to the chronification of pain after HZ.

## Pathophysiology

TG-PHN, caused by the VZV reactivation (Fig. 1A), involves intricate changes in pain signaling pathways, leading to heightened pain response through nociceptors sensitization, sensitivity due to local inflammatory mediators, augmented pain pathways excitability, and diminished inhibitory control (Fig. 1B–E). Given structural changes found in the TG, its afferent and efferent nerve, and even the trigeminal brainstem complex, “neuropathy” or “neuronopathy” may be better terms for TG-PHN [9, 18]. Investigating the multifaceted pathophysiological elements and their clinical correlations may offer valuable insights into more effective treatment approaches.

VZV replication can induce acute injury to sensory neurons, exacerbating inflammatory tissue damage (Fig. 1B). In various cell and tissue types, VZV, either directly or indirectly, triggers an increase in pro-inflammatory cytokines, such as interleukin (IL)-1β, IL-2, IL-6, IL-17, IL-18, and tumor necrosis factor-alpha (TNF-α) [43–46, 47•]. The presence of viral proteins coupled with immune responses initiates inflammation at the affected site and contributes to pain and heightened sensitivity [47•, 48]. Elevated serum IL-6 has been correlated with PHN pain severity, suggesting IL-6 is a potential diagnostic marker for PHN [49].

The intricate interplay between heightened inflammation and neuronal activity underscores the complexities of pain modulation [50]. Pro-inflammatory cytokines IL-1β and TNF-α play a pivotal role by sensitizing nociceptors, influencing pain signaling [51–53]. TNF-α activation can induce hyperexcitability through voltage-gated sodium channels [54, 55]. Voltage-gated ion channel expression alterations have been identified in secondary TN caused by herpes simplex virus type 1 [56]. Targeting voltage-gated sodium channels using lidocaine has emerged as an effective pain treatment strategy in PHN [57, 58].

Cellular remodeling through voltage-gated cation channels and G protein–coupled receptor modifications significantly affects pain transmission in PHN (Fig. 1B, C). Notably, calcium-permeable channels wield control over diverse intracellular calcium dynamics, with implications for various chronic pain disorders [59]. Investigating the role of the ATP purinergic (P2X7) receptor, a nonselective cation membrane pore channel, Zhu et al. found that inhibition of P2X7R diminished pain sensitivity and mitigated cellular stress and necrotic cell death processes, offering potential therapeutic avenues [60].

The role of calcium channels extends to NMDA receptor antagonists, a common therapeutic target for neuropathic pain conditions, including PHN. Activation of NMDA receptors contributes to mechanical allodynia, highlighting their pivotal role in this context [61]. Renewed interest in NMDA’s involvement in HZ and PHN pain stems from agents like ketamine, a selective antagonist of NMDA channels [62].

TG-PHN often results in hyperalgesia to noxious stimuli (e.g., heat, cold, mechanical pressure, and chemical), attributed to nociceptor transduction, which plays a central role in VZV-associated pain. Transient receptor potential (TRP) channels, such as TRPV1, transient receptor potential vanilloid 1 (a nonselective cation channel, which processes heat and capsaicin sensitivity), and TRPA1, transient receptor potential ankyrin 1 (uniquely sensitive to multimodal activation, including temperature, mechanical, oxidation, and several exogenous and endogenous compounds), play a crucial role in this process [63]. Capsaicin, a TRPV1 agonist, has long been a treatment for neuropathic pain, including PHN.

Calcitonin gene-related peptide (CGRP) has become a prime target for neuropathic pain and migraine management [64]. Skin biopsies of PHN patients have shown increased CGRP levels [65], and PHN patients have elevated serum CGRP levels compared to HZ patients without PHN (Fig. 1) [102]. It is notable that pharmacologic treatments targeted to neurotransmitter systems may have differential effects in different body regions. CGRP receptor antagonists work better in facial than lumbar experimental neuropathic pain; on the other hand, it was suggested that drugs primarily targeting nociceptive system sensitization (i.e., anti-inflammatories or ion channel modulators) may be more effective in thoracolumbar PHN than in the trigeminal PHN patients [29••].

The role of substance P (SP) and its G protein–coupled receptor NK-1R (neurokinin 1-receptor) is important in VZV-associated pain. VZV-infected human spinal astrocytes induced nuclear localization of NK-1R, associated with viral spread, and inhibition of NK-1R with the highly selective NK-1R antagonist, aprepitant, mitigated viral spread [66]. SP-NK-1R signaling is also important in immune cell signaling [67]. Additionally, aprepitant suppressed microglia activation and reduced inflammatory pain in mice [68]. Although the exact mechanisms of aprepitant’s potential efficacy in TG-PHN remain to be elucidated, its role as a potent treatment targeting inflammatory pain suggests promising avenues for investigation.

While VZV infection has conventionally been associated with peripheral effects, the presence of acute or persistent inflammation and nerve damage can lead to enduring modifications in central nervous system (CNS) pain pathways (Fig. 1D). Aδ and C fibers innervating the skin transmit somatic pain to the trigeminal brainstem nuclear complex, including the subnucleus caudalis (SNVc). Pain signals then undergo systemic modulation through diverse mechanisms. Inhibitory interneurons release neurotransmitters such as gamma-aminobutyric acid (GABA) and glycine to attenuate nociceptive signals via hyperpolarization or inhibition of nociceptive pathways. Conversely, excitatory TG neurons release neurotransmitters like glutamate and substance P to amplify pain transmission by depolarizing nociceptive pathways (Fig. 1C, D). Pain signals ascend to the thalamus and somatosensory cortex, leading to pain perception. Recent studies in a rat model of VZV-associated PHN found that VZV infection of the whisker decreased the pain threshold [69, 70]. Importantly, their results showed decreased GABA cell activity within the thalamus following VZV infection into the whisker pad, which induced orofacial pain behavior (Fig. 1E).

In summary, the cascading effects of VZV reactivation on the nervous system create the complex landscape of TG-PHN. The insights gleaned from the intertwined roles of cytokines, cation channels, TRP channels, and neuropeptides like CGRP and substance P illuminate potential avenues for more targeted interventions. Emerging agents offer promise in addressing the inflammatory component of TG-PHN. The equilibrium between excitation and inhibition within the CNS is paramount for effective long-term pain management. A potential explanation for PHN pain resolution in less than 1 year in most patients might be that the peripheral sensitization spontaneously resolves; it may also imply that there may be a window of opportunity during the first year after HZ, during which mechanisms underlying the PHN development might be controlled [12].

## Treatment of TG-PHN

PHN (especially TG-PHN) is difficult to manage and has no specific treatment, so prevention appears critical (Table 1).

**Table 1:** Treatment modalities for trigeminal postherpetic neuralgia

**Treatment**	**Examples**	**Pros**	**Cons**
Preventive			
* Zoster vaccine*	Zostavax (ZVL) Shingrix (RZV)	More than 60% effective to prevent PHN; 67% effective against HZO (RZV)	Lower and waning efficacy for ZVL, not in immunocompromised. Two doses and reactogenicity with RZV. Cost.
* Zoster treatment*	Antivirals, antiseizures, other pain interventions, including procedures	Symptomatic treatment of HZ, may shorten its burden and duration	Uncertain benefit to prevent PHN
Conservative			
* Pharmacologic*	ASDs: gabapentin, pregabalin, etc Antidepressants: TCAs, SNRIs Opioids: tramadol, etc	Easy to administer, can be continued after HR, can be combined	Multiple side effects: i.e., sedation, weight gain, dizziness, nausea, and constipation. Variable benefit.
* Topical*	Lidocaine, capsaicin, etc	Targeted. No systemic SE	Limited benefit. Local irritation
* Behavioral*	CBT, relaxation, sleep	Help coping with pain and mental health matters	Limited access. Limited benefit
* Acupuncture (A)*	Multiple modalities, i.e., ordinary A. and electro-A	Adjuvant for pain and anxiety relief, no SE	Limited access. Limited benefit
Interventional			
* Botulinum Toxin-A*	Sub-dermal injection in affected dermatome	Complex mechanism of action, minimally invasive	No standardized protocol. Face muscle weakness, ptosis, respiratory difficulty
* Peripheral nerve blocks*^a^	Injection of local anesthetic (+/− steroid) around TG, nerve, or nerve branch	Specific trigeminal nerve target, no systemic effects (unless steroid used)	Nerve injury. Systemic steroid effects or localized atrophy (if steroid used)
* Peripheral neurolysis*^a^	RFA, cryo- or chemical ablation of trigeminal nerve or branches.	Specific trigeminal nerve target, no systemic effects (unless w/ steroid)	Nerve injury. Systemic steroid effects or localized atrophy (if steroid used). Skin burns, pain flare. Persistent sensory deficit, anesthesia dolorosa
* Stellate ganglion block*^a^	Injection of local anesthetic in plane w/ Chassaignac’s tubercle	Reduces sympathetically mediated pain	Self-limited Horner syndrome. Phrenic nerve palsy. Injury to arteries, spinal nerves, brachial plexus)
* Neuromodulation (PNS, SCS)*^a^	Placement adjacent to specific trigeminal nerve branch or at cervicomedullary junction in epidural space	May address multiple pain pathways, long term, allow for program adjustments after implantation	Paresthesias. Procedural related complications (epidural hematoma, infection, damage to spinal cord, lead fracture)

^a^Limited evidence—Case reports, case series

*PHN* postherpetic neuralgia, *HZ* herpes zoster, *ASD* antiseizure drugs, *TCA* tricyclic antidepressants, *SNRI* serotonin and norepinephrine reuptake inhibitors, *CBT* cognitive behavioral therapy, *SE* side effects, *TG* trigeminal ganglion, *RFA* radiofrequency ablation, *SCS* spinal cord simulator, *PNS* peripheral nerve simulator

### Prevention

#### Vaccination

In clinical trials, the live attenuated vaccine ZVL (Zostavax) had 66% efficacy against PHN in individuals aged 60 and over, while the efficacy of the recombinant subunit vaccine RZV (Shingrix) against PHN was 76% overall and 88% in individuals aged 70 and older [2, 10]. The superior efficacy of RZV led to its preferred recommendation for adults ≥ 50 years old [10]. With an aging population, the incidence of HZ, particularly HZO, is rising [71]; it is estimated that, by 2050, in the absence of HZ vaccination, 20.7 million persons over 50 years of age will experience PHN globally, underscoring the urgency to develop and use vaccines to safeguard older adults from the complications of HZ [10, 72].

#### Treatment of Zoster Pain

Acute treatment of HZ, including antivirals, analgesics, and interventional treatments, may potentially prevent the development of PHN, but the evidence is weak [73, 74]. High-voltage long-duration pulse radiofrequency (PRF) neuromodulation of the Gasserian ganglion appeared to be effective for preventing PHN in the elderly [75].

### Pain Management-Conservative

Patients with PHN treated early (before 9 months) may have better outcome [76].

#### Systemic

Gabapentin (1800–3600 mg/day) and pregabalin (300–600 mg/d) (both FDA approved for PHN) are first line and probably act by modulating α2-δ site of voltage-gated calcium channels to decrease neurotransmitter release and excitatory pain signal transmission. In PHN, pregabalin seems better at alleviating pain and sleep than gabapentin, but the latter may be better tolerated [35]. Mirogabalin (15–30 mg/d), a new similar ligand, produced a significant dose-dependent reduction in the average daily pain score in patients with PHN (24% trigeminal) [77]. Tricyclic antidepressants (i.e., amitriptyline, nortriptyline, 50–150 mg/d each) are used off label for PHN but can be the first line, with the caveat of greater side effects in elderly. The use of serotonin and norepinephrine reuptake inhibitors (e.g., duloxetine, 60–120 mg/d; venlafaxine, 150–225 mg/d) or non-gabapentinoid antiseizure medications (carbamazepine 200–1200 mg/d, oxcarbazepine 600–1200 mg/d, lamotrigine 100–300 mg/d, valproic acid 500–1000 mg/d) is based on their benefit in other neuropathic pains including trigeminal neuralgia [78]. Note that target daily doses mentioned may not always be tolerated. A study investigating medical and pharmacy claims for PHN treatment identified that, overall, opioids were the most frequently prescribed initial treatment for PHN (21.6%), followed by gabapentin (15.1%), nonsteroidal anti-inflammatory drugs (NSAIDS) (8.9%), lidocaine patch (8.3%), pregabalin (3.3%), tricyclic antidepressants (TCAs) (2.5%), and capsaicin (< 1%) [79]. Even with the most effective medication, a significant response (> 50% reduction in pain) is achieved in less than 50% of patients [80]. Combination therapy is often necessary where monotherapy is insufficient or adverse effects limit dosing [74, 81, 82].

#### Local

Lidocaine is FDA approved for treatment of PHN as a 5% plaster, once daily. Capsaicin 8% patch single administration is also FDA-approved treatment for PHN, may repeat after 3 months, and was reportedly used successfully for TG-PHN [83]; caution required due to skin irritation. Evidence for low-dose capsaicin cream (0.025–0.075%) is inconclusive.

There is no definitive evidence for the efficacy of behavioral interventions in PHN, although it has been suggested they may be suitable for this condition and adjunct cognitive behavioral therapy may improve outcomes [84]. Acupuncture may reduce pain intensity in PHN, alleviate anxiety, and improve quality of life [85].

### Pain Management-Interventional

Pain refractory to conservative measures often warrants procedural intervention.

#### Botulinum Toxin-A Injection

Botulinum Toxin-A (BTX-A) is a potent neurotoxin that acts by complex mechanisms impeding impulse transmission at the motor and sensory nerve terminals. Its use for treating facial pain involves injections into the epidermis and dermis of the affected regions, approximately 1 cm apart, with 2–5 units deposited at each site, with the total dose varying between 25 and 200 units/session in studies. This regimen may be repeated at 8–12 weeks. The most common side effect is local pain, which usually resolves within hours. The use of BTX-A in treating PHN has been evaluated multiple times in double-blind randomized control trials [86, 87]. Each of these trials demonstrated a significant reduction in visual analog scale (VAS) pain scores (primary endpoint) in BTX-A groups out to 12 weeks compared to baseline, as well as increased sleep times (secondary endpoint). Future studies are recommended to identify the optimal dosing for standardization.

#### Nerve Block(s) and Nerve Ablation

Local anesthetic injections may provide relief in many patients and can be used as a diagnostic tool followed by nerve ablation or stimulation [88, 89]. The most common targets are the supraorbital, infraorbital, auriculotemporal, and mental nerves, but blocks of deeper branches, including the mandibular and maxillary nerves, can also be performed. Injections around targeted nerves (typically less than 3 ml of anesthetic, with or without steroid, using a small gauge needle, 27 or 30 g) can be performed safely and effectively in an outpatient setting using landmarks and/or ultrasound. Duration and degree of pain relief vary greatly, but if temporary symptomatic relief is achieved with nerve block, nerve ablation (radiofrequency or cryo-ablation) may be performed for longer-lasting relief. These procedures cause more discomfort during ablative stimulation, which often necessitates oral or intravenous sedation to complete the procedure safely and effectively. Given the location of these nerves in the subcutaneous layers of the face, pulsed RFA is often preferred over continuous delivery to reduce the risk of skin burns and damage to surrounding structures [90]. Additional to nerve ablation, perineural adjuncts such as dexamethasone can result in improved relief [91].

#### Stellate Ganglion Block

Stellate ganglion blocks (SGBs) are more frequently performed for acute HZ pain than TG-PHN. Symptomatic relief in patients with chronic TG-PHN pain varies, with some case reports citing complete symptom resolution for 6 months, while other small series report less pain relief (merely 0–50%) [92, 93]. SGBs are most commonly performed under ultrasound or fluoroscopic guidance by injecting local anesthetic at the level of the C6 vertebral body (vertebral artery safe within the foramen behind Chassaignac’s tubercle) anterior to the prevertebral fascia and longus colli muscle and posterior to the carotid sheath. The most sensitive indicator of successful block is temperature change in the ipsilateral upper extremity.

#### Spinal Cord Stimulator (SCS)

Implantation of SCS for treatment of TG-PHN is undergoing early investigation, with no multi-center prospective trials completed to date. The principles underlying the role of SCS in trigeminal pain reduction relate to its ability to attenuate nociceptive reflexes and cause broad inhibition of sensory afferent inputs through stimulation of non-nociceptive A-β fibers; however, unintentional stimulation of surrounding structures may occur. Placement of SCS leads at the cervicomedullary junction, with direct access via the epidural space, targets the trigeminal sensory tract and pars caudalis. Several small, single-institutional studies, some dating back more than 30 years, have described high technical and procedural success rates with such implantation and reported achieving significant pain relief in > 70% of patients beyond 12 months follow-up [94–96]. The most frequent reason for failed trials prior to implantation was facial paresthesias.

#### Peripheral Nerve Stimulator (PNS)

In 2014, the Neuromodulation Appropriateness Consensus Committee included PHN in its evidence review and summary of indications, specifically mentioning that stimulators were effective for treating TG-PHN [97]. The use of PNS offers a targeted and less invasive neuromodulation approach than the high cervical SCS leads implantations. Supra- and infraorbital nerves are the trigeminal branches most amenable to PNS. Candidates for PNS include patients with medically intractable neuropathic pain, with some preservation of sensation to the affected region [98]. Diagnostic nerve blocks with local anesthetic are performed for confirmation of specific nerve involvement prior to trial or permanent implant. The trial leads can be placed using ultrasound or fluoroscopy. The trial goal is > 50% pain relief, typically lasts 5–14 days to evaluate continued pain relief and allows familiarization with the device’s function and paresthesias. After a successful trial, the permanent leads are anchored and then tunneled posterolaterally to be terminally connected to the implanted generator. While much of the published literature focuses on refractory TN, many reports include subsets of patients with TG-PHN. Multiple small series have reported nearly all patients experiencing symptom relief at > 6 months follow-up, a reduction in concomitant opioid use, and improved quality of life [99–101]. Patients who did not respond well to trial stimulation were most commonly having V3 distribution continued pain. There are difficulties in obtaining good mandibular coverage with PNS, and these patients are often referred for high cervical SCS implants.

## Conclusions

As science continues to untangle the intricate web of clinical, cellular, and molecular events underlying VZV-associated pain, the prospects for enhanced pain management strategies and improved patient outcomes are ever more promising. Understanding the pathogenesis of TG-PHN’s is imperative to overcoming the challenges of its clinical management. Future research endeavors should focus on unraveling the complexities of the interplay between peripheral and central mechanisms, paving the way for more precise and efficacious treatments for this debilitating condition.

## Data Availability

No original research data was included. All authors are available for any questions.
